# Strategic Feeding of Ammonium and Metal Ions for Enhanced GLA-Rich Lipid Accumulation in *Cunninghamella bainieri* 2A1

**DOI:** 10.1155/2014/173574

**Published:** 2014-06-03

**Authors:** Shuwahida Shuib, Wan Nazatul Naziah Wan Nawi, Ekhlass M. Taha, Othman Omar, Abdul Jalil Abdul Kader, Mohd Sahaid Kalil, Aidil Abdul Hamid

**Affiliations:** ^1^School of Biosciences and Biotechnology, Faculty of Science and Technology, Universiti Kebangsaan Malaysia (UKM), 43600 Bangi, Selangor, Malaysia; ^2^Faculty of Science and Technology, Universiti Sains Islam Malaysia, Bandar Baru Nilai, 71800 Nilai, Negeri Sembilan, Malaysia; ^3^Department of Chemical and Process Engineering, Faculty of Engineering, Universiti Kebangsaan Malaysia (UKM), 43600 Bangi, Selangor, Malaysia

## Abstract

Strategic feeding of ammonium and metal ions (Mg^2+^, Mn^2+^, Fe^3+^, Cu^2+^, Ca^2+^, Co^2+^, and Zn^2+^) for enhanced GLA-rich lipid accumulation in *C. bainieri* 2A1 was established. When cultivated in nitrogen-limited medium, the fungus produced up to 30% lipid (g/g biomass) with 12.9% (g/g lipid) GLA. However, the accumulation of lipid stopped at 48 hours of cultivation although glucose was abundant. This event occurred in parallel to the diminishing activity of malic enzyme (ME), fatty acid synthase (FAS), and ATP citrate lyase (ACL) as well as the depletion of metal ions in the medium. Reinstatement of the enzymes activities was achieved by feeding of ammonium tartrate, but no increment in the lipid content was observed. However, increment in lipid content from 32% to 50% (g/g biomass) with 13.2% GLA was achieved when simultaneous feeding of ammonium, glucose, and metal ions was carried out. This showed that the cessation of lipid accumulation was caused by diminishing activities of the enzymes as well as depletion of the metal ions in the medium. Therefore, strategic feeding of ammonium and metal ions successfully reinstated enzymes activities and enhanced GLA-rich lipid accumulation in *C. bainieri* 2A1.

## 1. Introduction

*C. bainieri* 2A1 is an oleaginous fungus isolated from Malaysian soil. This fungus has the capability of producing up to 30% lipid (g/g biomass) containing 10–15% GLA [1]. Lipid produced in some oleaginous fungi contains large amount of high-valued essential polyunsaturated fatty acids (PUFAs) such as GLA and arachidonic acid (AA) [2]. GLA is an important intermediate in the biosynthesis of biologically active prostaglandin that is derived from linolenic acid. However, GLA cannot be synthesized by human and therefore must be consumed in the daily diet. In Europe, GLA is known as “King's Cure-All” because of its nutritional effects to cure diseases such as decreasing blood cholesterol, suppressing acute and chronic inflammations, and improving atopic eczema [3].

Previously, GLA is commercially produced from the seeds of Evening Primrose (*Oenothera biennis*), Borage (*Borago officinalis*), and Blackcurrant (*Ribes nigrum*) [4]. However, there are a number of problems associated with mass production of GLA from these seeds such as low productivity, long duration of crop cultivation, and requirement of huge area for harvesting [5]. Therefore, to overcome these problems, zygomycetes such as* Cunninghamella*,* Mucor,* and* Mortierella* are often mentioned in the literature as alternatives of GLA producers due to their desirable characteristics such as high productivity of the GLA, short process cycle, and easier scalability. Currently, sugar-based low-cost materials and several types of residues (e.g., glycerol, lignocellulosic sugars, cheese whey, molasses, tomato waste hydrolysates, etc.) have been successfully considered as substrates for the production of SCO by zygomycetes. Glycerol is a major by-product discharged after the biodiesel manufacturing process has been used as a substrate for SCO production in* C. echinulata* and* M. isabellina* [6]. Besides, Vamvakaki et al. [7] reported that* Mortierella isabellina* had an outstanding performance in biomass, lipid, and GLA production when grown on cheese whey. Moreover,* Cunninghamella echinulata *and* M. isabellina* showed capability of using molasses for SCO production due to its high sugar content [8]. Tomato waste hydrolysate also has been applied as a substrate for SCO production in* C. echinulata* [9]. Among the above mentioned low-cost waste materials, lignocellulose is of great importance because of its continuous supply as a result of land cultivation. As xylose is the second most abundant sugar of lignocellulosic biomass, growth of* C. echinulata* and* M. isabellina* on xylose containing nitrogen-limited media resulted in the accumulation of significant quantities of lipid as well as GLA [6].

Lipid accumulation in oleaginous microorganisms is triggered by a nutrient imbalance in the culture medium. When nitrogen sources are depleted, excess carbon in the medium continues to be assimilated by the cells and converted into storage lipid [10]. Several studies have shown that media with varying composition of trace elements affect growth and lipid accumulation in various fungal species. In relation to lipid and PUFAs production, Mg^2+^, Mn^2+^, Fe^2+^, Ca^2+^, Cu^2+^ and Zn^2+^ have been shown to influence lipid and AA as well as GLA production by* Mortierella rammanniana var rammaniana* [11].

Oleaginous microorganisms are capable of accumulating large amount of lipid because of their ability to produce a sufficient supply of NADPH and a continuous supply of acetyl-CoA which is a necessary precursor for lipid biosynthesis [12]. The lipid biosynthesis pathway in oleaginous microbes occurs due to the concerted action of a few key lipogenic enzymes, such as ME, FAS, and ACL [10]. It has been suggested that ME specifically functions as the sole provider of NADPH, which is required for FAS activity in* Mucor circinelloides* and* Mortierella alpina*. Thus, ME is implicated as the key enzyme in regulating the extent of lipid accumulation [13]. ACL is a cytosolic enzyme that catalyzes the cleavage of citrate to generate acetyl CoA, which can be used as a precursor for lipid biosynthesis [14, 15]. On the other hand, FAS catalyzes the synthesis of palmitate, in which acetyl-CoA and malonyl-CoA are substrates and NADPH is the reducing agent. Other than these key lipogenic enzymes, NAD : ICDH was reported as being an important enzyme involved in the initiation of lipid accumulation. It was proposed that the activity of this enzyme would be inhibited after nitrogen depletion, as a result of significant decrease in AMP concentration via the activation of AMP deaminase; its activity is dependent on AMP concentration. This would then result in the accumulation of citrate which serves as the precursor for ACL which generates acetyl-CoA, a precursor for lipid synthesis [13].

Our previous observations showed that* C. bainieri* 2A1 is capable of accumulating up to 30% lipid (g/g biomass) containing 10–15% GLA [1]. However, cessation of lipid accumulation occurred after 48 hours of growth, although glucose was still present in the medium [16]. Further analysis showed that cessation of lipid accumulation coincides with a significant decrease in the specific activities of ME, FAS, and ACL which suggest that diminishing enzymes activities is the probable cause which leads to the cessation of lipid accumulation. Although this has also been reported to occur in the other oleaginous fungi such as* M. circinelloides* and* M. alpina* [13], no further evidence is currently reported. Our preliminary studies have also shown that an initial concentration of metal ions in the medium affects lipid accumulation as well as lipogenic enzyme activities [17]. As they are important in lipogenesis, the metal ions probable involvement in contributing to the enhancement of lipid accumulation should be carried out. Therefore, in this study, the effect of reinstating the activities of ME, FAS, and ACL as well as the possible involvement of metal ions in increasing lipid accumulation in* C. bainieri *2A1 was investigated.

## 2. Materials and Methods

### 2.1. Microorganism

*C. bainieri* 2A1, a local soil isolate, was obtained as a stock culture from the School of Biosciences and Biotechnology, Faculty of Science and Technology, Universiti Kebangsaan Malaysia, Bangi, Selangor. The cultures were maintained at 4°C on Potato Dextrose agar (PDA), and subculture was performed every 2 months.

### 2.2. Preparation of Media and Culture Conditions

A nitrogen-limited medium [18] containing the following constituents (g/L): (NH_4_)_2_C_4_H_4_O_6_ 1.0, KH_2_PO_4_ 7.0, Na_2_HPO_4_ 2.0, MgSO_4_·7H_2_O 1.5, yeast extract 1.5, CaCl_2_ 0.1, FeCl_3_·6H_2_O 0.008, Co(NO_3_)_2_·6H_2_O 0.0001, ZnSO_4_·7H_2_O 0.0001, CuSO_4_·5H_2_O 0.0001, and MnSO_4_·5H_2_O 0.0001 was sterilized at 121°C for 40 min. Glucose (30 g/L) was added separately after sterilization. Seed culture was prepared by transferring spore suspension into 500 mL shake flasks containing 200 mL of nitrogen-limited medium to a final concentration of 1 × 10^5^ spore/mL. The cultures were incubated at 30°C and agitated at 200 rpm for 48 h. Ten percent (v/v) of the culture was then used for subsequent inoculations. All experiments were carried out using 500 mL conical flasks containing 200 mL of the nitrogen-limited medium as described above. Cultivation was carried out at 30°C, with agitation at 200 rpm for 120 h. For fed-batch experiments, simultaneous feeding of concentrated ammonium tartrate, glucose, and each of the metal ions (Mg^2+^, Mn^2+^, Fe^3+^, Cu^2+^, Co^2+^, Ca^2+^, and Zn^2+^) was carried out at 72 h to reach their initial concentrations. Controls that consisted of cultures fed with glucose and either the metal ions or ammonium tartrate were also conducted. Cultures were sampled every 24 h and assayed for enzyme activities (ME, FAS, and ACL), glucose, ammonium, biomass concentrations, lipid content, and concentration of metal ions in the biomass and culture broths. Fed-batch experiments were also conducted in 5 L fermentor (Minifors, Switzerland) equipped with an online data acquisition and control system. Culture pH and dissolved O_2_ were monitored with a pH meter (Mettler-Toledo, Switzerland), and oxygen probe (Mettler-Toledo, Switzerland). The cultivation conditions were as follows: 10% (v/v) seed culture, temperature of 30°C, pH 6 (by automating control using 5 M NaOH), agitation rate at 600 rpm, aeration rate at 0.8 v/v/m, and dissolved oxygen at 40%–50% saturation. The initial culture contained 4 L nitrogen-limited medium.

### 2.3. Analytical Methods

Ammonium concentration was determined using the indophenol method [19], while glucose concentration was determined using a glucose oxidase GOD-PERID test kit (Boehringer Mannheim) according to the manufacture's instruction. A 1 mL of culture medium was filtered using Whatman nylon membrane filter with pore size of 0.45 *μ*m and was diluted. Then, quantification of metal ions concentration in the medium was performed using inductively coupled plasma mass spectrometry (ICP-MS) (Perkin-Elmer Elan 5000) [20]. Fungal biomass was harvested by filtration of 200 mL culture through pre-weight Whatman No. 1 filter paper followed by washing with 400 mL distilled water. The filtered mycelia were then freeze-dried overnight to a constant weight. Dried mycelia were then ground into a powder using pestle and mortar, followed by lipid extraction using chloroform/methanol 2 : 1 (v/v) [21]. The mixture was filtered through Whatman No. 1 filter paper to remove cell debris and the organic fractions, pooled in separating funnel, and washed with 1% sodium chloride then with distilled water using separating funnel. The chloroform extract was rotary evaporated. The remaining lipid was dissolved in 3 mL diethyl ether and transferred to pre-weight vial and the diethyl ether evaporated at room temperature under the hood. The sample was dried in desiccator for 24 h and weighted. Lipid content was expressed as % (g/g of biomass). Fatty acid methyl esters (FAMES) were analyzed using a Shimadzu GC-2010 FID. Separation was achieved by using a packed column (DB-23) and flame ionization detector (FID). The column was maintained at 250°C. The fatty acids composition presence in the sample was calculated based on the peak area of corresponding methyl ester against reference standard of FAME mixture. All data presented were the mean values of triplicates and the standard deviation was not more than 20%.

### 2.4. Production of Cell-Free Extract

Harvested mycelia were suspended in 20% (w/v) extraction buffer containing 100 mM KH_2_PO_4_/KOH (pH 7.5), 20% (v/v) glycerol, 1 mM benzamidine, 1 mM mercaptoethanol, and 1 mM EDTA and were disrupted using pestle and mortar. The cell suspension was centrifuged at 10 000 g for 15 minutes at 4°C, and the resulting supernatant, termed the cell-free extract (cfe), was used for enzyme assays. Protein concentration was determined using the method of Bradford [22] with BSA as a standard.

### 2.5. Enzyme Assays

The activities of ME, FAS, and ACL were determined using continuous assays following the oxidation or reduction of NAD(P)(H) at A_340nm_ and carried out at 30°C [23–25]. The change in absorbance was followed continuously for 15 min using software (UV PROBE 2.31). Specific activity is expressed as nmol/min·mg protein.

## 3. Results and Discussion

### 3.1. Relationship between Lipid Accumulation and ME, FAS, and ACL Specific Activity in* C. bainieri* 2A1

**Figure 1:**
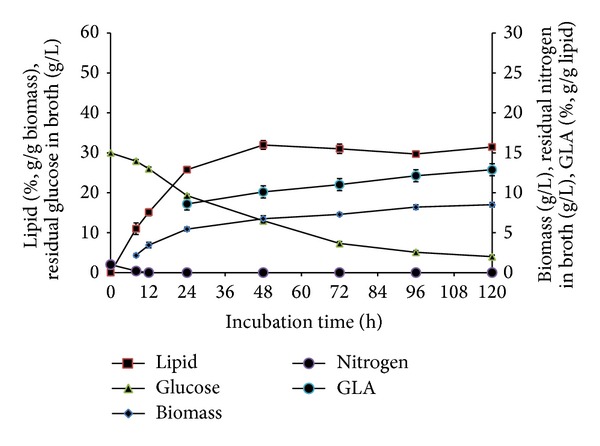
Profile of growth and lipid accumulation of* C. bainieri* 2A1. The culture was cultivated in 500 mL shake flask containing 200 mL of nitrogen-limited medium at 30°C, 200 rpm, and starting inoculums of 10% (v/v). The data represent means ± SDs, *n* = 3.

*C. bainieri* 2A1 showed similar lipid accumulation and ME, FAS, and ACL profiles as reported in other filamentous fungi in batch cultivation [13]. Lipid accumulation was initiated after total depletion of ammonium at 12 hours of cultivation with lipid content showing an increase from 12% to 32% (g/g biomass) at 48 h with 10.1% GLA (g/g lipid) (Figure 1). However, lipid accumulation ceased after 48 hours of cultivation although excess glucose was still present in the medium. This observation was similar to various reports involving filamentous fungi such as* M. circinelloides*,* M. alpina* [13], and* M. isabellina* [26, 27]. Conversely, in* C. echinulata* lipid accumulation was significantly delayed and was reported to probably be due to delayed decrease in the activity of NAD- and NADP isocitrate dehydrogenase [28] though the concentration of intracellular AMP was not mentioned to relate with the activity of the enzyme present. Diminishing activity of NAD : ICDH through repression or significant decrease in intracellular AMP concentration via the activation of AMP deaminase would result in the accumulation of citrate which serves as the precursor for ATP citrate lyase (ACL). In* C. bainieri,* NAD : ICDH activity decreased after N limitation but remained detectable until 96 h (results not shown), whereas NADP : ICDH remained active during lipogenesis until the end of cultivation [16]. This was similar to what was reported in* M. circinelloides* and* M. alpina, *where the activity of NAD : ICDH was detectable until 120 hours of cultivation. However, the activities in* M. alpina* decreased more rapidly which corresponds to its higher lipid level achieved [13].

**Figure 2:**
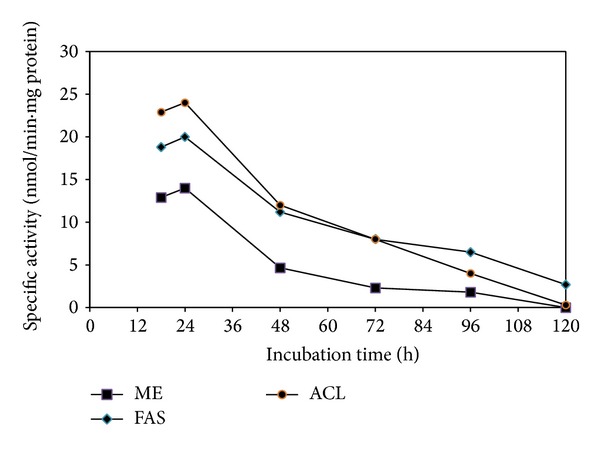
Specific activity of ME, FAS, and ACL of* C. bainieri* 2A1. The culture was cultivated in 500 mL shake flask containing 200 mL of nitrogen-limited medium at 30°C, 200 rpm, and starting inoculums of 10% (v/v). The data represent means ± SDs, *n* = 3.

When the specific activities of ME, FAS, and ACL of the culture throughout the cultivation period were investigated, results showed that the highest specific activities of ME, FAS, and ACL were detected at 24 hours of cultivation (12.3, 21.1, and 24.7 nmol/min·mg protein, resp.). However, the activities of ME, FAS, and ACL markedly decreased at 48 h and this coincided with the cessation of lipid production with percentage of reduction of the activity of 73%, 52%, and 64%, respectively, and diminished at 120 h (Figure 2). This suggests the existence of vital relationship between the specific enzymes activities (ME, ACL, and FAS) and lipid accumulation. At least two other oleaginous zygomycetes (*M. circinelloides* and* M. alpina*) were reported to show corresponding decline in lipid production with decreasing ME, FAS, and ACL activity [13]. As the reintroduction of limited concentration of ammonium has been shown to reinstate the activity of ME, FAS, and ACL in* M. circinelloides* and* M. alpina* [13], effects of feeding of ammonium tartrate at 72 h on the activities of the enzymes were investigated.

### 3.2. Effects of Feeding of Ammonium Tartrate on ME, FAS, and ACL Specific Activity and Lipid Accumulation in* C. bainieri* 2A1

**Figure 3:**
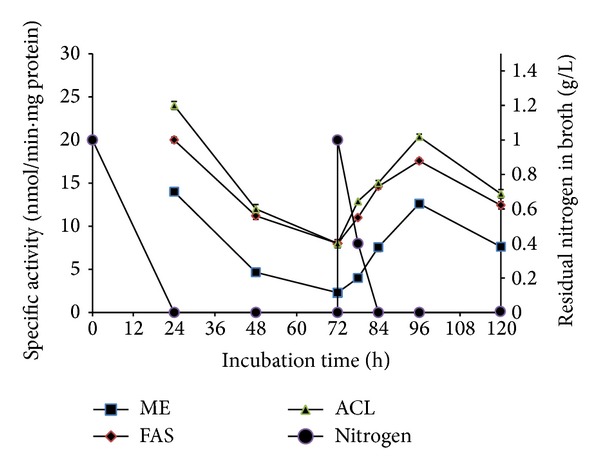
Effect of feeding ammonium tartrate at 72 hours of cultivation on ME, FAS, and ACL specific activities of* C. bainieri* 2A1. The culture was cultivated in 500 mL shake flask containing 200 mL of nitrogen-limited medium at 30°C, 200 rpm, and starting inoculums of 10% (v/v). The data represent means ± SDs, *n* = 3.

Results showed that the specific activities of ME, FAS, and ACL increased significantly from 2.3 to 12.6, from 8.0 to 17.3, and from 7.9 to 20.3 nmol/min·mg protein, respectively, within 24 h after feeding of ammonium tartrate and decreased at 120 hours of cultivation (Figure 3). Ammonium is not a positive effector for these enzymes in this fungus as no significant increase in the specific activities of ME, FAS, and ACL was observed when ammonium tartrate was incorporated into the assay mixtures. This suggests that the increase in specific activities after the feeding is more likely a result of an increase in the synthesis of the enzymes due to the availability of ammonium as the nitrogen source. Although reinstatement of ME, FAS, and ACL activities was achieved, no increment in lipid content was observed, where the lipid content remained at 31% (g/g biomass). As glucose concentration at the point of feeding was low, the experiment was repeated with simultaneous feeding of concentrated ammonium tartrate and glucose to reach their initial concentrations (1 and 30 g/L, resp.) at 72 hours of cultivation. However, similar results were observed where the specific activities of the three enzymes increased but with no increase in lipid content (data not shown). These results indicate that lipid accumulation stopped although the cultures were in the most optimal condition for lipid accumulation, that is, limited N, excess C, and in the presence of ME, FAS, and ACL activities.

**Figure 4:**
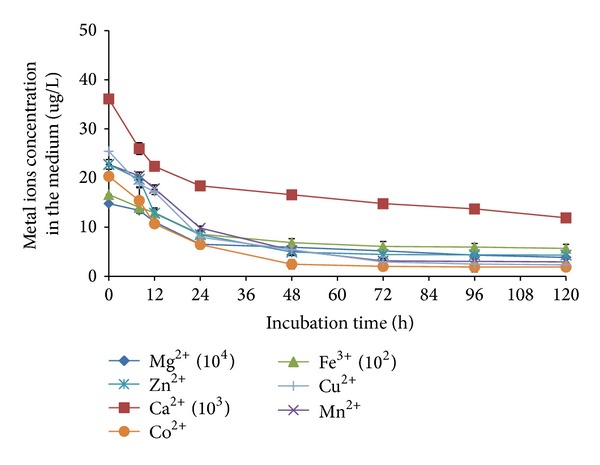
Concentration of metal ions left in culture medium throughout the cultivation of* C. bainieri* 2A1. The culture was cultivated in 500 mL shake flask containing 200 mL of nitrogen-limited medium at 30°C, 200 rpm, and starting inoculums of 10% (v/v). The data represent means ± SDs, *n* = 3.

As the limitation of lipid accumulation occurred at the latter stage of the cultivation, it was thought that the limitation could be due to the depletion of components of the medium such as metal ions. According to Dyal et al. [11], Mg^2+^, Mn^2+^, Fe^2+^, Ca^2+^, Cu^2+^, and Zn^2+^ have been shown to influence lipid accumulation in* Mortierella rammanniana var rammaniana. *Previous work also showed that different initial concentrations of Mg^2+^, Fe^3+^, and Zn^2+^ have significant effects on lipid accumulation in* C. bainieri* 2A1 [17]. It was thought that the possible limiting concentration of these metal ions in the medium contributed to the limitation of lipid production despite the fact that the successful reinstatement of the enzymes was achieved. When analysis of the medium throughout the cultivation using ICP-MS was carried out, a pronounced decrease in the concentrations of each of the metal ions (Mg^2+^, Mn^2+^, Ca^2+^, Cu^2+^, Fe^3+^, Co^2+^, and Zn^2+^) was observed, though at varying rates, thus supporting the suggestion (Figure 4). Most of the metal ions (Fe^3+^, Mg^2+^, Mn^2+^, Cu^2+^, Co^2+^, and Zn^2+^) diminished within 24 h soon after nitrogen limitation, whereas Ca^2+^ was still present at approximately 50% of its initial concentration at 120 h. To the best of the authors' knowledge, the trend and relationship of utilization of metal ions during growth and lipid accumulation phase have not been reported. Therefore, to further establish the significance of the activities of ME, FAS, and ACL as well as the depletion of metal ions in determining the extent of lipid production in* C. bainieiri* 2A1, the effects of simultaneous reinstatement of the activities of the enzymes and replenishment of metal ions in the medium on lipid production were carried out.

### 3.3. Possible Involvement of Metal Ions in the Limitation of Lipid Accumulation

When simultaneous feeding of ammonium tartrate, glucose, and all metal ions (Mg^2+^, Mn^2+^, Fe^3+^, Cu^2+^, Ca^2+^, Co^2+^, and Zn^2+^) was carried out at 72 h, reinstatement of the enzyme activities was followed by an increase in lipid content from 32% to 50% (g/g biomass) with 13.2% (g/g lipid) of GLA content at 120 h (Table 1). Total of dry biomass and lipid yields which were 0.34 and 0.17 g gram of glucose consumed, respectively, were similar to* M. isabellina* grown on high sugar content media [26, 27]. On the other hand, in* C. echinulata *ATHUM 4411 that was grown on glucose and tomato waste hydrolysate, the concentration of GLA produced per liter of culture medium was reported to be 0.8 and 0.78 g/L, respectively [9, 26, 27]. Interestingly, in our present works, a much higher concentration of GLA was obtained, 0.97 g GLA per liter of culture medium. To the best of the authors' knowledge, this amount is amongst the highest achieved compared to other zygomycetes such as* M. circinelloides, M. rammanniana, M. isabellina,* and* C. echinulata* which commonly produced between 0.2 to 0.8 g GLA per liter of culture medium [26, 27]. On the contrary, no increment in lipid content was observed when the enzymes activities were not reinstated (by feeding of metal ions or glucose only) or when the reinstatement of the enzymes activities was performed with the omission of metal ions during the feeding. These results showed that the cessation of lipid accumulation observed previously was as a result of diminishing activities of the enzymes as well as the depletion of metal ions.

**Figure 5:**
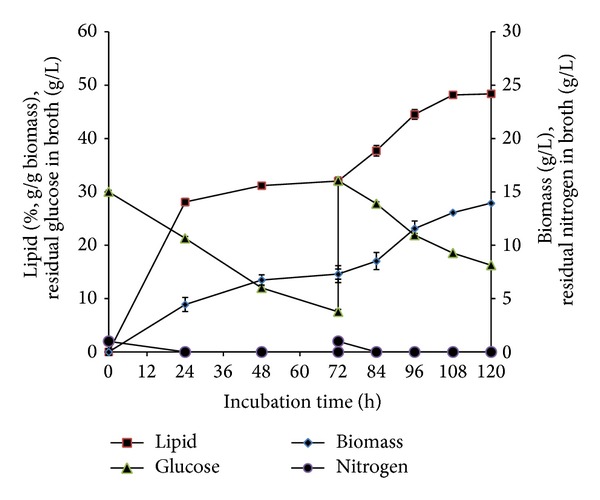
Effect of feeding ammonium tartrate, glucose, and Fe^3+^ at 72 h in lipid accumulation of* C. bainieri* 2A1. The culture was cultivated in 500 mL shake flask containing 200 mL of nitrogen-limited medium at 30°C, 200 rpm, and starting inoculums of 10% (v/v). The data represent means ± SDs, *n* = 3.

**Figure 6:**
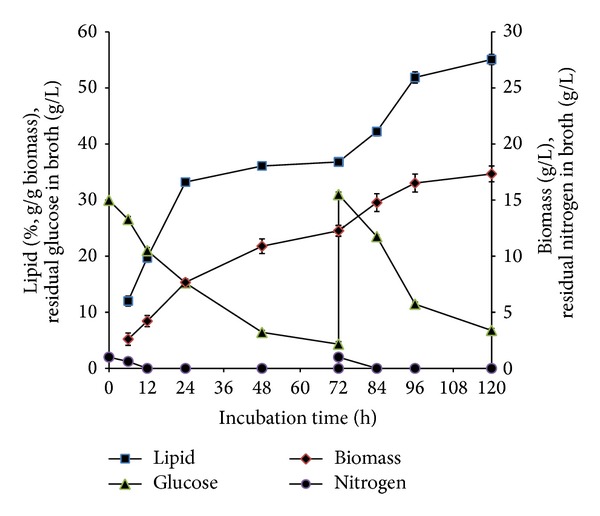
Effect of feeding ammonium tartrate, glucose, and Fe^3+^ at 72 h in lipid accumulation of* C. bainieri* 2A1. The culture was cultivated in a 5 L fermenter with 4 L of nitrogen-limited medium with 30°C, pH 6.00, aeration 0.8 v/v/m, agitation 600 rpm, and starting inoculums of 10% (v/v). The data represent means ± SDs, *n* = 3.

When further experiments were carried out by simultaneous feeding of ammonium tartrate, glucose with individual ions, that is, either Fe^3+^, Mg^2+^, Mn^2+^, or Zn^2+^, similar increment of lipid content (from 32% to up to 48%, g/g biomass) was observed with the GLA content between 10 and 15% (g/g lipid) (Table 1). The highest lipid and GLA yields were achieved when Fe^3+^ was fed compared to other metal ions (0.15 and 0.02 g, resp., per gram of glucose consumed) (Figure 5). The metal ions such as Cu^2+^, Co^2+^, and Ca^2+^ also have been tested alone for the lipid and GLA production. However, no increment in lipid content was observed when these metal ions were fed into the culture and also in controls, which were fed with the omission of either the metal ions or ammonium tartrate. Metal ions play important roles in the biological function of many enzymes as the presence of the metal ions is crucial for the activity of some enzymes. These enzymes are known as metalloenzymes. Therefore, lipid accumulation is more pronounced in the presence of metal ions such as Fe^3+^ because the ions may serve as a cofactor for the key lipogenic enzymes such as ME and ACL. In addition, cultivation of* C. bainieri* 2A1 in 5 L fermentor with the same feeding strategy showed that lipid accumulation was enhanced from 48% (g/g biomass) (in shake flaks) to 55% (g/g biomass) (Figure 6). This result was similar to* M. isabellina* cultivated in 3 L fermentor with glucose as a substrate where increment in lipid content was observed following scale up of the process from 9.9 g/L of lipid in shake flask to 12.7 g/L of lipid in 3 L fermentation setup [8]. Conversely, in* Thamnidium elegans, *better results were reported in shake flasks compared to larger fermenter setup [29]. It is noteworthy to point out that the increment of lipid content achieved after feeding at 72 h was not followed by any decrease in GLA content, the effect commonly observed in oleaginous fungi when increment in lipid accumulation is achieved through increased glucose concentration [9, 26, 27, 30, 31].

In oleaginous microorganisms, lipid turnover typically occurred when carbon source was exhausted in the culture medium. Papanikolaou et al. [28] reported that repression of lipid turnover could be due to multiple limitation factors such as the nitrogen and several microelements like Mg^2+^, Fe^3+^ or elements derived from the yeast extract, whilst lipid turnover was activated when metal ions were added. However, no proof of lipid turnover was observed in* C. bainieri* 2A1 as the result showed constant lipid content from 48 h onwards, in the presence of glucose (Figure 1). This result was similar to at least two other zygomycetes:* M. circinelloides* and* M. alpina* [15]. In our experiments, glucose was always kept over 10 g/L and lipid turnover was not observed in this fungus, although metal ions were added (Figure 4). As proved by our data, lipid synthesis was reinitiated when metal ions were added in the culture whereby multiple limitations resulted in the cessation of lipid accumulation in* C. bainieri* 2A1. Therefore, these results showed that, as reported by Papanikolaou et al. [28], presence of metal ions is vital for lipid synthesis as well as lipid turnover for zygomycetes. In addition, the cessation of lipid accumulation at 48 h in* C. bainieri* 2A1 was not caused by saturation of lipid accumulated inside this fungus as reported to occur in* C. echinulata* and* M. isabellina* [28]. This is because the increment of lipid content in* C. bainieri* 2A1 was observed only when feeding of ammonium, glucose, and metal ions was carried out at 72 h.

In addition, fatty acid compositions such as C16:0 (palmitic acid), C18:0 (stearic acid), C18:1 (oleic acid), C18:2 (linoleic acid), and C18:3n-6 (*γ*-linolenic acid) were analyzed at the end of the cultivation period (120 h) by gas chromatography (Table 2). The results showed that C18:1 was the predominant fatty acid accumulated by this fungus in all experiments performed. It was followed by the amount of C16:0, whereas low content of C18:0 and C18:2 (12.03% and 8.78%, g/g lipid, resp.) was observed. The GLA composition was found in significant amount (12%, g/g lipid) in all experiments performed. These profiles were different in comparison to lipid production by several oleaginous fungi. Previous reports showed that C18:1 was the main fatty acid in* M. isabellina* when grown on high sugar content medium. However significant amount of C16:0 and C18:2 (22% and 18%, g/g lipid, resp.) but with low amount of C18:0 and C18:3n-6 was reported [26, 27]. In* C. echinulata* ATHUM 4411 that was grown on tomato waste hydrolysate with the feeding of glucose, after 12 days of cultivation, the concentration of GLA fell dramatically from 25% (before the feeding) to 12% (g/g lipid) with the increment of the concentrations of C18:1, C16:0, and C18:0 being reported [9]. Therefore, generally, the differences in fatty acid composition could be related to strains, fermentation time, initial sugar concentration, types of carbon and nitrogen sources used, and initial molar of C/N ratio.

## 4. Conclusions

Cessation of lipid accumulation in* C. bainieri* 2A1 is caused by diminishing activities of ME, ACL, and FAS as well as depletion of metal ions in the medium. Simultaneous feeding of ammonium (which resulted in the reinstatement of the activities of the enzymes) and metal ions at 72 h successfully reinstated the enzymes activities and enhanced lipid accumulation, followed by consistent percentage of GLA content throughout the cultivation of* C. bainieri* 2A1.

## Figures and Tables

**Table 1:** Effect of feeding on lipid and GLA production. Cultures were fed with several medium components at 72 hours of cultivation. Lipid was extracted and analyzed for GLA content at 120 hours of cultivation. The data represent means ± SDs, *n* = 3.

Feeding	Biomass, X (g/L)	Lipid, L % (g/g biomass)	GLA % (g/g lipid)	Yield of biomass per glucose consumed, Y_X/G_	Yield of lipid per glucose consumed, Y_L/G_	Yield of GLA per glucose consumed Y_GLA/G_
Ammonium tartrate, glucose, and all metal ions (Mg^2+^, Mn^2+^, Fe^3+^, Cu^2+^, Ca^2+^, Co^2+^ and Zn^2+^)	14.77 ± 0.45	50.95 ± 1.07	13.24 ± 0.13	0.34	0.17	0.02

Ammonium tartrate, glucose, and Fe^3+^	13.95 ± 0.60	48.75 ± 0.67	12.66 ± 0.05	0.31	0.15	0.02

Ammonium tartrate, glucose, and Mn^2+^	12.05 ± 0.42	44.22 ± 0.80	11.86 ± 0.32	0.22	0.13	0.01

Ammonium tartrate, glucose, and Mg^2+^	13.28 ± 0.40	39.24 ± 0.71	12.13 ± 0.39	0.32	0.13	0.01

Ammonium tartrate, glucose and Zn^2+^	11.04 ± 0.55	37.73 ± 0.54	11.45 ± 0.27	0.26	0.09	0.01

Ammonium tartrate and glucose	12.57 ± 0.54	31.57 ± 0.79	12.19 ± 0.49	0.34	0.11	0.01

Without feeding	8.42 ± 0.58	31.64 ± 0.88	12.95 ± 0.14	0.33	0.31	0.01

**Table 2:** Fatty acid composition of the cellular lipids of *C. bainieri* 2A1 at 120 hours of cultivation.

Feeding	Fatty acid composition (%)				
	C16:0	C18:0	C18:1	C18:2	C18:3 n-6
Ammonium tartrate, glucose, and all metal ions (Mg^2+^, Mn^2+^, Fe^3+^, Cu^2+^, Ca^2+^, Co^2+^, and Zn^2+^)	19.95	12.03	30.66	8.78	13.24

Ammonium tartrate, glucose, and Fe^3+^	21.35	8.95	35.47	9.77	12.66

Ammonium tartrate, glucose, and Mn^2+^	19.21	9.75	37.17	9.69	11.86

Ammonium tartrate, glucose, and Mg^2+^	27.87	12.99	32.03	4.29	12.13

Ammonium tartrate, glucose, and Zn^2+^	21.47	12.71	42.75	7.83	11.04

Without feeding	18.62	10.27	40.35	15.72	12.95

## Abbreviations

PUFAs: Polyunsaturated fatty acids
SCO: Single cell oil
GLA: *γ*-linolenic acid
AA: Arachidonic acid
NADPH: Nicotinamide adenine dinucleotide phosphate
AMP: Adenosine monophosphate
Acetyl Co-A: Acetyl co-enzyme A
ME: Malic enzyme
FAS: Fatty acid synthase
ACL: ATP citrate-lyase
NAD : ICDH: Nicotinamide adenine dinucleotide : isocitrate dehydrogenase
N: Nitrogen
C: Carbon
X: Biomass (g/L)
L: Lipid.
